# Invasive Respiratory Fungal Infections in COVID-19 Critically Ill Patients

**DOI:** 10.3390/jof8040415

**Published:** 2022-04-17

**Authors:** Francesca Raffaelli, Eloisa Sofia Tanzarella, Gennaro De Pascale, Mario Tumbarello

**Affiliations:** 1 Dipartimento di Scienze di Laboratorio e Infettivologiche, Fondazione Policlinico Universitario A. Gemelli IRCCS, 00168 Roma, Italy; francesca.raffaelli@policlinicogemelli.it; 2 Dipartimento di Scienze Biotecnologiche di Base, Cliniche Intensivologiche e Perioperatorie, Università Cattolica del Sacro Cuore, 00168 Roma, Italy; eloisasofia.tanzarella@policlinicogemelli.it (E.S.T.); gennaro.depascale@policlinicogemelli.it (G.D.P.); 3 Dipartimento di Scienze Dell’emergenze, Anestesiologiche e Della Rianimazione, Fondazione Policlinico Universitario A. Gemelli IRCCS, 00168 Roma, Italy; 4 Dipartimento di Biotecnologie Mediche, Università degli Studi di Siena, 53100 Siena, Italy; 5 UOC Malattie Infettive e Tropicali, Azienda Ospedaliero-Universitaria Senese, 53100 Siena, Italy

**Keywords:** COVID-19, SARS-CoV-2, respiratory fungal infection, CAPA, pneumocystosis, CAM

## Abstract

Patients with coronavirus disease 19 (COVID-19) admitted to the intensive care unit (ICU) often develop respiratory fungal infections. The most frequent diseases are the COVID-19 associated pulmonary aspergillosis (CAPA), COVID-19 associated pulmonary mucormycosis (CAPM) and the *Pneumocystis jirovecii* pneumonia (PCP), the latter mostly found in patients with both COVID-19 and underlying HIV infection. Furthermore, co-infections due to less common mold pathogens have been also described. Respiratory fungal infections in critically ill patients are promoted by multiple risk factors, including epithelial damage caused by COVID-19 infection, mechanical ventilation and immunosuppression, mainly induced by corticosteroids and immunomodulators. In COVID-19 patients, a correct discrimination between fungal colonization and infection is challenging, further hampered by sampling difficulties and by the low reliability of diagnostic approaches, frequently needing an integration of clinical, radiological and microbiological features. Several antifungal drugs are currently available, but the development of new molecules with reduced toxicity, less drug-interactions and potentially active on difficult to treat strains, is highly warranted. Finally, the role of prophylaxis in certain COVID-19 populations is still controversial and must be further investigated.

## 1. Introduction

Severe acute respiratory syndrome coronavirus 2 (SARS-CoV-2) is the causative agent of the recent pandemic of coronavirus disease 19 (COVID-19). COVID-19 primarily affects the upper respiratory tract and ranges from asymptomatic or mildly symptomatic infection to lower tract damage, leading to severe bilateral pneumonia [1]. Patients with severe illness can develop respiratory failure with arterial hypoxemia and respiratory distress, needing intensive care unit (ICU) admission and invasive respiratory support [2].

ICU length of stay and duration of mechanical ventilation are usually prolonged, due to the extensive lung damage caused by the virus and to the high risk of secondary infections. The suggested pathophysiologic mechanism is based on the capability of SARS-CoV-2 to impair immune response against microbial agents, altering the dynamics of inter-microbial interactions, and promoting the proliferation of pathogenic species [3]. Further factors contributing to invasive respiratory fungal infections are poor health conditions of the ICU patients and concomitant therapies with corticosteroids and immune-modulating agents such as anti-interleukin-6 monoclonal antibodies [4,5].

Since the pandemic outbreak of COVID-19 there have been many reports of fungal coinfections in critically ill patients without immunological disorders, mainly due to respiratory invasive fungal infections, including COVID-19-associated pulmonary aspergillosis (CAPA) and *Pneumocystis jirovecii* pneumonia (PCP) [6]. In the beginning, the reports have been based on anecdotal cases, observational studies and autoptic findings [7,8,9,10], followed by more extensive and coordinated multicentric investigations [5,11,12,13].

Other invasive respiratory fungal infections have been reported worldwide in COVID-19 patients, including mucormycosis, cryptococcosis, fusariosis, histoplasmosis and other endemic mycoses. When these infections involve critically ill patients, they are usually associated with a relevant severity degree, contributing to the high mortality rate of such population [6].

## 2. COVID-19 Associated Pulmonary Aspergillosis (CAPA)

Determining the incidence of CAPA is particularly problematic, due to the lack of an endorsed case definition, especially in the beginning of the pandemic. The most frequently applied classification is the one published by Koehler et al. in the ECMM/ISHAM consensus criteria [4], combining host factors, clinical features and mycological evidence. A recent review by Feys et al. [14] summarized data of 7047 patients from 48 studies, reporting the incidence of CAPA until 12 October 2021. Since then, only few observational trials or case series have been published [15,16,17,18,19,20,21,22]. The reported CAPA incidence among hospitalized patients with severe COVID-19 varies widely, ranging from 1 to 42% [23,24], with the four largest multicenter prospective cohort studies reporting rates of 15%, 28%, 14% and 2.5%, respectively [5,22,25,26]. Only in a few cases was CAPA proven, while the majority had a probable or possible diagnosis. Most patients developed CAPA between day 4 and 11 after ICU admission, undergoing microbiological diagnostic work-up due to their clinical condition worsening. However, the only study that involved routine bronchoscopy on day 0 and day 7 of ICU admission was the one of Bartoletti et al., reporting 14 of 108 (13%) patients with a GM index >1 at ICU admission [5,27]. An increased mortality in patients with proven, probable or possible CAPA has been widely reported: as noted by Feys et al. in a recent review analyzing 728 CAPA, all-cause death rate was 55.2%, significantly higher than patients without invasive aspergillosis [14].

The severity of clinical status on ICU admission, quantified trough severity scores such as APACHE II, SAPS II and SOFA, appear to be associated with CAPA, as reported by several cohort studies [13,14,20,25,28]. Despite this, there is no clear association with length of ICU stay, duration of mechanical ventilation, need for veno-venous ECMO support and poor PaO2/FiO2 ratio [4,13,14,15,26,29,30,31].

Interestingly, some authors argued that the increased risk of secondary infections due to corticosteroids could be related not merely to their immunosuppressive effects, but also to the difficulties in managing steroid-induced hyperglycemia, that could undermine the positive immunomodulatory effects of the therapy [32,33]. However, many experts do not recommend the discontinuation of corticosteroids in case of diagnosis of CAPA, raising many concerns about the increased risk of developing this complication in such patients [13,15,25,26,27].

The same applies to IL-1 and IL-6 inhibitors [34,35,36] where the impact of immunomodulation on both innate and adaptative immune responses may increase patients’ susceptibility to invasive fungal diseases. This assumption seems to be corroborated by some of the studies with the highest incidence of CAPA, where up to 28% of patients receiving intravenous or subcutaneous tocilizumab developed pulmonary aspergillosis [5]. In line with the current evidence, a recent multicenter observational study on a cohort of more than 500 COVID-19 patients, showed a strong association of higher SAPS II value and the use of dexamethasone with the probability to develop CAPA [37].

The diagnosis of invasive fungal infections in ICU patients with SARS-CoV-2 infection and acute respiratory failure is challenging and requires the integration of clinical, radiological and microbiological aspects that are not always easy to detect.

The clinical presentation of CAPA may be variable and should be suspected in case of refractory fever or the onset of fever after a period of 48 h of defervescence during appropriate antibiotic therapy, worsening respiratory status, tachypnoea or increasing oxygen requirements, haemoptysis, chest pain and pleural friction rub in patients with refractory respiratory failure despite receiving all support recommended for patients with COVID-19 [4].

**Figure 1:**
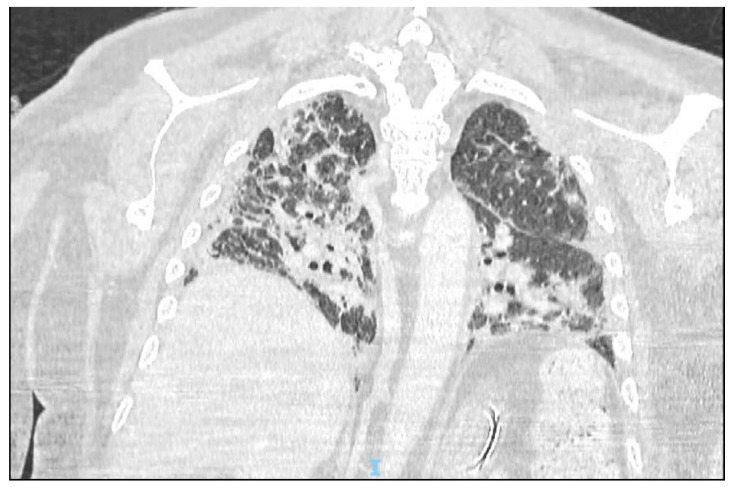
*Aspergillus niger* co-infection in COVID-19 ARDS. Bronchoalveolar lavage, soon after endotracheal intubation, showed galactomannan positivity (OI = 5) and direct identification of the mold. The patient already received IL-6 inhibitors and was ongoing dexamethasone. The clinical picture healed after four weeks of voriconazole.

Radiology plays an important role in the evaluation of invasive fungal infections even though is not to be considered a totally reliable criterion for the diagnosis. Some COVID-19 radiological findings are consistent with the typical lesions of CAPA and are not easy to be recognized, especially in patients with ARDS (Figure 1). The typical early radiological findings of COVID-19 in patients includes in early stages peripheral, bilateral ground-glass opacities, consolidation or crazy-paving; instead, at late stages typical findings are ground-glass opacities with mixed consolidation and reverse halo sign or other findings of organizing pneumonia [38]. Ghazafari et al. reported that there was no significant difference between the group of COVID-19 patients with and without invasive mold infection (total 105 patients) with respect to radiological findings and more than a half of the cases had bilateral ground-glass opacities and/or consolidation extension, which could only be attributable to COVID-19 [39]. Another study reported that the halo sign, a typical aspergillosis radiologic finding in neutropenic patients, has also been reported in patients with COVID-19, without aspergillosis, which may be an expression of the vascular injury and the microthrombosis, peculiar features of COVID-19 [40]. Combined pulmonary aspergillosis and mucormycosis in patients with COVID-19 are described in a case report [41]; CAPA and COVID-19 associated pulmonary mucormycosis (CAPM) indeed share the same risk factors and the imaging findings are overlapping [42,43]. In this case report, chest CT revealed extensive bilateral pneumonia and the development of bilateral upper lobe cavitations, consistent with both infections [41].

In addition to clinical and radiological aspects, microbiological evidence from specific respiratory samples provides more specific data for the diagnosis of CAPA. During the first wave of COVID-19 pandemic, the use of bronchoscopy to obtain bronchoalveolar lavage specimen for the microbiological detection of invasive fungal infections was discouraged for the risk of contagiousness for health-care workers from procedures that generate aerosol exposure [44,45]. A recent study reports that bronchoscopy can be safely performed in COVID-19 patients when strict personal protection equipment (PPE) is applied [46,47,48,49]. Bronchoscopy is recommended in patients with suspected co-infection, to allow airway visualization, bronchial alveolar lavage fluid and biopsy which remains the gold standard for diagnosis of invasive aspergillosis and IATB [4]. A reduction in bronchoscopy performance led to an increased use of alternative non-bronchoscopic bronchial lavage (NBL) specimens, including sputum, bronchial aspirates (BA) and tracheal aspirates (TA) for which there are no validation of *Aspergillus* biomarkers, resulting in increased difficulty in distinguishing between airway colonization and invasive infection. In a study on 63 COVID-19 patients Galactomannan antigen (GM) on TA samples resulted in false positives [50], although an increased specificity of GM on TA may be achieved with a higher positivity cut off of 2.0 [51]. Instead, Van Grootveld described a concordance between culture and PCR of 88% for both TA and BAL [50]. Serum GM detection, indicative of angioinvasive disease and with poor sensitivity when testing in non-neutropenic patients [52], is observed in only 20% patients with CAPA [11,14]. Recently, a study evaluated mycological criteria (direct examination, culture, PCR, galactomannan serum and on respiratory samples, 1,3-β-D-glucan and plasma PCR) suggesting using a criteria combination to increase the possibility to identify patients with CAPA [53]. The wide range of incidence of CAPA (1–42%) is probably due to several factors such as the distinction between colonization and infection, made challenging and confounding by the use of 1,3-β-D-glucan and galactomannan for non-neutropenic patients, such as COVID-19 patients.

Studies and the case reports published so far on CAPA have had to address the difficulty of diagnosis of this disease using different criteria and diagnostic algorithms. The definition of invasive fungal disease provided through the criteria of the European Organization for Research and Treatment of Cancer (EORTC)/Mycosis Study Group Education and Research Consortium (MSGERC) categorized into proven, probable and possible invasive fungal disease in patients with high-risk conditions (i.e., immunodepression especially due to hematologic malignancy). This definition is rarely applicable to COVID-19 critically ill patients in ICU that usually do not have specific host factors that classically predispose to invasive fungal infections [54,55].

In 2012, a specific diagnostic algorithm, AspICU, was validated for ICU patients that allows to diagnose and discriminate the *Aspergillus* spp. colonization from invasive pulmonary infection, particularly difficult in this category of patients. According to the AspICU algorithm, putative IPA is defined by the presence of compatible clinical signs and symptoms, abnormal lung imaging by X-ray or CT, and either a lower respiratory tract specimen positive for *Aspergillus* or a host risk factor. In the absence of one of these criteria, the patient is classified as colonization [56]. The case series have used the modified AspICU, that includes among microbiological criteria the detection of serum and bronchoalveolar lavage GM with a positivity cut-off 0.5 and 1.0, respectively [57].

Before the CAPA consensus criteria were defined, an expert opinion on IAPA in ICU patients proposed a CAPA definition in which the entry criterion was pulmonary infiltrates, not attributed to another cause, with at least one mycological finding (positive serum GM > 0.5 or BAL ≥ 1), positive *Aspergillus* culture of BAL, or, if BAL is not performed, sputum or TA, or cavitation in an area of pulmonary consolidation patients [58]. Bartoletti et al. applied this CAPA definition to intubated COVID-19 patients and probable CAPA was diagnosed in 27.7% patients. They concluded that the use of the newly proposed CAPA criteria may allow earlier diagnosis than AspICU criteria and might prioritize prompt antifungal treatment [5].

In 2020, the European Confederation for Medical Mycology (ECMM) and the International Society for Human and Animal Mycology (ISHAM) proposed consensus criteria for the definition of CAPA providing three different grades (possible, probable and proven CAPA) to homogeneously classify patients in registries and clinical trials and to identify patients in clinical settings [4]. Proven CAPA requires a demonstration of invasive growth of *Aspergillus* species in tissue or sterile sites. Factors that differentiate the new definition of probable and possible CAPA mainly concern the presence of host factors, the radiological findings, the diagnostic specimens and the microbiological tests. In particular, these consensus definitions also include in the “possible” diagnosis of CAPA patients who have not been subjected to bronchoscopy, and therefore, have upper respiratory tract samples (NBL) evidence of aspergillosis. Moreover, this is the first ICU specific definition that include PCR on BAL and serum as microbiological criterion for the diagnosis of probable CAPA [4]. Recently, an international experts’ report stated that bronchoscopy with BAL remains the cornerstone of CAPA diagnosis and positive *Aspergillus* culture from BA or TA sample are to be considered triggers to perform bronchoscopy and BAL [12].

Several recent studies show that the evidence of invasive fungal infections in critically ill COVID-19 patients is associated to increased mortality rates [5,26] and early diagnosis allows early antifungal treatment. Some authors have shown that a percentage of patients with CAPA survived without antifungal therapy; this may be due to the difficult to distinguish between invasive disease and colonization of aspergillosis [5]. The taskforce report recommends antifungal therapy in patients with positive BAL *Aspergillus* culture, GM and/or *Aspergillus* PCR and confirmed IATB [27].

At this time, there are no data that suggest that the treatment of CAPA should be different than that of invasive aspergillosis (Table 1). The ECMM/ISHAM consensus recommend the use of voriconazole or isavuconazole as first-line antifungal therapy for possible, probable and proven CAPA [4]. Although voriconazole is the recommended first-line treatment for IPA, including severely critically ill patients in intravenous administration [59], there are some considerations for voriconazole use in critically ill COVID-19 patients. Given the well-known drug–drug interactions due to being a substrate for CYP2C19, CYP2C9 and CYP3A4, voriconazole interacts with COVID-19 therapy, such as remdesivir, which is also metabolized via CYP3A4 [4]. Isavuconazole is the primary alternative treatment option due to the favorable pharmacokinetic profile and the reduced toxicity [60]. Although isavuconazole is a substrate for CYP3A4 too, the drug–drug interactions are less pronounced than voriconazole [4]. Posaconazole was recently shown to be non-inferior to voriconazole for the treatment of invasive pulmonary aspergillosis [61], although the real-life data of the use of posaconazole, as well as isavuconazole, in ICU patients with aspergillosis is limited.

Liposomial amphotericin B is a broadly effective alternative treatment option for aspergillosis, although the nephrotoxicity that in ICU patients, often affected by renal insufficiency, complicates starting or requires discontinuation of this antifungal drug [62]. This is particularly relevant for patients infected by SARS-CoV-2 which has shown renal tropism and been described as a frequent cause of kidney injury [63].

Echinocandins are not considered first-line treatment options for aspergillosis in monotherapy for their limited antifungal activity against *Aspergillus* spp. [64,65], furthermore they can be considered as salvage therapy and in association with an azole might have some therapeutic advantage in critically ill patients [66,67]. A combination of echinocandin and voriconazole or amphotericin B may be used to treat CAPA in areas of high prevalence of azole resistance strains, until the susceptibility become available [68,69].

New antifungal classes are under development (fosmanogepix, ibrexafungerp, opelconazole and olorofim) [70] and may become future options with good efficacy without the drug–drug interactions and toxicity [4,71].

The optimal duration of CAPA therapy is unknown, but the expert panel suggests 6–12 weeks as a treatment course and suggest including a follow-up lung CT imaging to document the resolutions of the infiltrates before termination of treatment [4]. Follow-up GM-index in serum to consider the therapeutic response might be limited by its poor sensitivity when testing serum in non-neutropenic patients. Instead, follow-up GM-index in respiratory specimens could be useful to assess the efficacy in patients who are at the beginning GM positive, which may also give indications on the duration of therapy [4].

The therapeutic drug monitoring in order to ensure the adequate triazole exposure should be performed for patients with CAPA in ICU that often have high variability to drug exposure due to impaired renal or hepatic function, renal replacement therapy or extracorporeal membrane oxygenation, alterations in protein binding [4,27,72]. Moreover, dexamethasone used in COVID-19 patients with pneumonia is a CYP450 enzymes inducer and cause a reduction of voriconazole plasma concentration [73,74]. The ECMM/ISHAM consensus recommend weekly therapeutic drug monitoring on patients with CAPA in cases of fully susceptible *Aspergillus* species, specifically for voriconazole and posaconazole. No isavuconazole target concentration has been defined, but therapeutic drug monitoring might be warranted in patients who are on renal replacement therapy and patients with obesity [4].

In patients with CAPA in ICU it is recommended to assess the concomitant corticosteroids therapy; in a recent review, Verweij et al. suggest the continuation of the dexamethasone therapy for the recommended time frame, if possible, and consider stopping corticosteroids when there is no clear hyperinflammation anymore, when it was given for 10 days and/or when there is evidence of angioinvasive CAPA [27].

The high prevalence of invasive fungal infections and the mortality rates in ICU patients as reported in literature may justify clinical trials evaluating antifungal prophylaxis in patients with COVID-19 and acute respiratory failure, similar to those proposed for IAPA [75]. A recent observational study comparing patients with or without antifungal prophylaxis with respect to CAPA incidence and mortality, shows that antifungal prophylaxis, mainly posaconazole, was associated with significantly reduced CAPA incidence, but no difference in mortality was observed [76]. One retrospective single-center case series from Belgium has reported the successful use of prophylaxis in terms of CAPA case reduction with inhaled liposomal Amphotericin B in a cohort of ICU patients with severe COVID-19 [77]. However, these data are derived only from observational and retrospective studies. Therefore, further study, especially prospective clinical trials, are warranted to evaluate the efficacy and safety of antifungal prophylaxis with respect to CAPA incidence and clinical outcomes, investigating also novel long-active antifungal, rezafungin, that could be a suitable alternative for this application [78].

## 3. COVID-19 Associated Pulmonary Mucormycosis (CAPM)

The majority of non-*Aspergillus* respiratory fungal infections reported were COVID-19 associated pulmonary mucormycosis (CAPM), complicating 0.15% of COVID-19 cases in a multicenter study from India [79], with a higher incidence in the subgroup of patients admitted to the ICU (1% in a multicenter study from France) [25]. The current estimated pooled prevalence of CAPM is of 5 per 10,000 patients hospitalized with COVID-19 [42]. CAPM accounts for about 9.5% of all cases of COVID-19 associated mucormycosis (CAM), although these numbers can underestimate the real burden of the problem according to the expert [42]. Many case reports and case series were published during the astonishing outbreak of the second wave that affected India in the first half of 2021: among them, *Rhizopus* was the predominant genus followed by *Mucor* and *Lichteimia* [80,81]. Most patients developed mucormycosis between day 8 and 20 after hospital admission, and in some cases, it was a necropsy finding [79,80,82,83,84].

Mortality rates of CAM are highly variable, with a lower rate in cases reported from India (36.5%) than from elsewhere (62%), probably due to the predominance of the rhino-orbital type in India, that is commonly associated with a better clinical outcome [79,80,81,82,85,86,87,88]. A French multicenter study reported that 30% of CAM patients died before the diagnosis was made and did not receive any treatment; the global mortality was 88% at week 12. This mortality rate might be partly explained by the higher frequency of pulmonary or disseminated diseases, which are classically associated with a poorer prognosis [89].

Hyperglycemia and uncontrolled diabetes, often secondary to corticosteroid therapies, are the most described risk factors and have a strong association with occurrence of mucormycosis [79,80,82,83,84,90,91]. This is due to the inhibition of many mechanisms of the host immune response, such as hyperglycemia-induced diabetic ketoacidosis (DKA), that leads to increased levels of free iron in the host and high ferritin blood levels. Indeed, it has been demonstrated that ferritin, along with promoting the growth of fungi, is also a strong marker of disease severity in COVID-19 patients and a key mediator of immune dysregulation, contributing to the cytokine storm and to the harmfulness of COVID-19 disease [33,87]. CAPM has been diagnosed more frequently in patients in immunosuppressive therapy for malignancy or organ transplantation [80].

A recent Delphi consensus statement from Fungal Infection Study Forum and Academy of Pulmonary Sciences defined the guidance for definition management and diagnosis of CAPM. Pulmonary mucormycosis occurring within 3 months of COVID-19 diagnosis was labelled CAPM and classified further as proven, probable, and possible [42].

Recently a review identified 180 cases of CAM, of which 14 CAPM cases reported were identified [82]. Pulmonary CT scan in suspected CAPM is recommended and the most frequent findings were consolidations and cavitation, pleural effusion, nodules, “reverse halo sign”, vessel occlusion or ground glass opacities, although these findings can also be found in patients with COVID-19 [43,82]. Interestingly, also rare but severe complications such as pulmonary artery pseudoaneurysm have been reported [92].

The different imaging features of CAPM were classified as highly suggestive, suggestive, non-specific, or not suggestive: the presence of a thick-walled cavity, reversed halo sign, large consolidation or necrotising pneumonia, mycotic aneurysm, bird’s nest sign, multiple large nodules, serial imaging showing cavity with an air-fluid level were considered as highly suggestive of CAPM [42].

Diagnostic confirmation of CAPM can be obtained by direct microscopy, culture, biopsy or molecular methods on clinical samples [42,93]. The consensus recently recommended early flexible bronchoscopy in most patients with CAPM for the visualization of airway abnormalities, performing endobronchial biopsies, and providing samples representing the lower respiratory tract (bronchoalveolar lavage or bronchial washings) [42].

The detection of circulating *Mucorales* DNA (cmDNA) has shown high sensitivity confirmed by a recent prospective trial that demonstrated sensitivity of 85.2% and specificity of 89.8% and positive and negative likelihood ratios 8.3 and 0.17, respectively, suggesting the use in the diagnosis and follow-up after treatment initiation [93,94,95]. Since serum tests to detect *Mucorales* antigens are not available, cmDNA could be considered as a screening tool for COVID-19 patients allowing earlier diagnosis of invasive CAM [96].

Dual infections of CAPA and CAPM are described [41] and the diagnosis is challenging on the grounds that the imaging findings of CAPM overlap with CAPA [42,43]. The consensus recommends in patients with radiological features highly suggestive of CAPM, to continue the evaluation of CAPM despite diagnostic evidence of CAPA (microbiological or serological), in order to arrange antifungal therapy active against both *Aspergillus* and *Mucorales* and evaluate the need for surgery [42].

First line treatment with liposomal amphotericin B is recommended across all patterns of organ involvement, included pulmonary, although the known drug nephrotoxicity, especially in critically ill COVID-19 patients, often required dose adjustment or discontinuation. Isavuconazole and posaconazole are recommended as an alternative for the treatment of mucormycosis, especially if pre-existing renal compromise, and may be considered as step-down therapy once the disease is controlled and the susceptibility is confirmed [42]. In parallel to antifungal treatment, surgical debridement of the primary focus should be performed when feasible (Table 1) [42,93]. The optimal duration of primary therapy for CAPM is unclear; the experts recommended that the duration of therapy be based on response assessment, generally achieved by 4–6 weeks of primary therapy [42]. Regarding CAPM prevention, the most important step is the appropriate use of glucocorticoids and other immunosuppressants for COVID-19 and the control of underlying risk factors (i.e., strict glycaemic control) is crucial also in order to improve outcomes in CAPM [42,97]. The expert panel advised against using antifungal prophylaxis for preventing CAM or CAPM in patients with COVID-19 [42].

## 4. Pneumocystis Jirovecii Pneumonia (PCP)

Although the incidence of coinfections by *Pneumocystis jirovecii* during COVID-19 is far below CAPA, *Pneumocystis jirovecii* pneumonia has been increasingly described, especially in patients with concomitant diagnosis of human immunodeficiency virus (HIV).

Casalini et al. reported twenty cases of PCP until October 2021, with 30% of patients with underlying HIV infection [6]. A total of 3 observational studies [11,98,99], 1 case series [100] and 2 case reports [101,102] described 33 other PCP coinfections: overall frequency of positive *Pneumocystis jirovecii* PCR findings ranged from 1.4% to 9.3% [103,104,105]. Interestingly, a review of 12 cases by Chong et al. reported a similar mortality rate between the HIV and non-HIV group in COVID-19 patients with *Pneumocystis jirovecii* coinfection (43% vs. 40%), with an overall mortality rate of 41.6% [106].

Lymphocytopenia, ARDS, steroids and immunomodulatory therapies are also well-known susceptibility factors for developing PCP. Even before the outbreak of COVID-19, some investigations reported high incidence of PCP in immunocompromised patients without HIV, due to the increasing number of patients receiving corticosteroids or immunosuppressive medications for autoimmune diseases, stem cell or solid organ transplantations [55]. Chong et al. recently described a cohort of COVID-19 patients with PCP: independently from the HIV status, they showed severe lymphocytopenia (<1000 cells/mm^3)^, with CD4+ cell count <200 cells/mm, all receiving long-term immunosuppressive agents and requiring invasive mechanical ventilation [106].

The diagnosis is mainly based on the evidence of *Pneumocystis jirovecii* DNA in respiratory samples. The high-resolution CT images play a relatively marginal role. The presence of cysts or fine reticular changes on CT scan are in favor of pneumocystosis, but ground-glass opacities pattern with interlobular septal thickening are common chest CT findings in PCP and COVID-19 [98,107]. In critically ill COVID-19 patients in whom a diagnostic bronchoscopy with a BAL cannot be safely performed, clinical and radiological features with elevated serum biomarker levels of lactate dehydrogenase (LDH) and 1,3-β-D-glucan may be the only useful tools to initiate empirical treatment [106,108]. As for aspergillosis, the distinction between infection and colonization with *P. jirovecii* is challenging. A study reports that 9% of critically ill patients with COVID-19 had a positive PCR on bronchial alveolar lavage [104], but PCR’s high sensitivity may lead to overestimate the diagnosis of *P. jirovecii* infection in colonized patients. The serum fungal marker 1,3-β-D-glucan is a helpful tool for the diagnosis of PCP [109], especially for its negative predictive value [104,110]. In addition, in COVID-19 patients with pneumonia, the use of corticosteroids may increase the difficulty and delay the diagnosis of PCP because their conditions may improve due to the well-known beneficial effect of steroids in severe PCP.

Trimethoprim-sulfamethoxazole (CTX), in combination with corticosteroids in severe disease, represent the recommended first-line treatment for PCP (Table 1) [111]. Some studies described improvement without therapy in COVID-19 patients, owing to the difficult discrimination between colonization and infection [99,104]. Pentamidine is considered a second-line choice when CTX is contraindicated. PCP primary chemoprophylaxis with CTX can be considered in selected high-risk COVID-19 patients being treated with a high steroid dosage, but further studies are needed [100].

## 5. Cryptococcosis

Fourteen cases of cryptococcosis in COVID-19 patients have been described to date, almost all from non-European countries: 9 from the USA and the others from Qatar, India, Brazil, Canada, Germany and Spain [112,113,114,115,116,117,118,119]. On two of them, *Cryptococcus* was primarily detected in respiratory samples, respectively, in the bacterial BAL culture plate [114] and in the biopsy of the lung lesion [119].

Diagnosis of cryptococcosis in patients without typical risk factors (i.e., HIV, transplantation) are often missed or significantly delayed and the sensitivity of cryptococcal antigen lateral flow assay (LFA) in serum, usually very high compared with standard cultures and serological diagnostic approach [120], is lower than that in HIV patients [121]. The sensitivity of serum cryptococcal antigen is unknown in COVID-19 patients with an impaired immune response. As such, the risk of dissemination is increased compared to immunocompetent patients; therefore, necessitating an evaluation for meningoencephalitis in COVID-19 patients with serologic or microbiologic evidence of cryptococcosis [112].

Fluconazole is the recommended antifungal treatment of pulmonary cryptococcosis, itraconazole, voriconazole and posaconazole are acceptable alternatives in mild-to-moderate disease. For severe or disseminated disease, liposomial amphotericin B plus flucytosine is recommended (Table 1) [122].

## 6. Other Invasive Respiratory Fungal Infections

Coinfections due to less common mold pathogens, such as the *Fusarium*, *Scedosporium* and endemic mycoses (*Coccidioides*, *Histoplasma*), have been increasingly reporting in the last two years, especially from non-European countries. Disseminated infections due to such mold pathogens usually occur in neutropenic or severely immunocompromised patients. Anyway, just like *Aspergillus* although to a lesser extent, they have been observed in a certain number of COVID-19 immunocompetent patients, with a wide spectrum of clinical manifestations.

Most of such cases are reported in patients with at least one comorbidity (hypertension, diabetes) and treated with corticosteroids during the hospitalization. Conversely a weaker association has been observed with anti-IL6 or anti-IL-1 drugs and HIV infections [118,123,124,125,126,127].

*Fusarium* and *Scedosporium* belong to a heterogeneous group of filamentous molds defined by the presence of hyaline hyphae on microscopic examination of tissue specimens (hyalohyphomycosis). Seven cases of pulmonary fusariosis (one *Fusarium proliferatum* in France, three *Fusarium incarnatum*, one *Fusarium fujikuroi*, one *Fusarium equiseti* and one *Fusarium solani* in Iran) and one case of lung infection due to *Scedosporium* (Chile) have been to date reported in the medical literature [39,128,129].

Diagnosis and management of those hyalohyphomycosis have been comprehensively dealt in the ESCMID and ECMM joint guidelines released in 2014 [130] and to date there are no further recommendations to be applied to COVID-19 patients. Radiological findings of pulmonary *Fusarium* and *Scedosporium* infections are often non-specific and similar to COVID-19 and *Aspergillus* related typical lesions. Importantly some case reports of *Fusarium* infection may not indicate pulmonary disease [39] but rather colonization; pulmonary fusariosis, like other mold infection, requires a predisposing risk factor and consistent imaging findings for the correct diagnosis [131]. The 1,3-β-D-glucan and galactomannan test are often positive, but not enough specific to discriminate between different fungal infections. The diagnosis requires culture identification of the mold from infected sites, while molecular-based identification appears promising but is still not fully standardized. Blood cultures may be positive in >50%, due to *Fusarium* and *Scedosporium* propensity to hematogenous spread [130].

Voriconazole represents the first-line treatment in *Scedosporium* and *Fusarium* infections (recommendation AII), while amphotericin B deoxycholate, liposomal amphotericin B formulations and various combinations, e.g., with caspofungin [132], can also be used in immunocompromised patients with fusariosis (BII). Surgical resection is recommended if the lesions are localized in both cases [130].

In the framework of the endemic mycoses, one case of coccidiomycosis, four cases of pulmonary histoplasmosis (two in Brazil, one in India, one in USA) and five cases of disseminated histoplasmosis (two in Argentina, one in USA, two in Brazil) were documented, half of them with a concomitant HIV infection [118,123,124,125,126,127,133,134,135].

Discussing the diagnostic tools and therapeutic management of endemic mycoses, we refer to most current guidelines published in 2021 by the ECMM with the International Society for Human and Animal Mycology [136].

Patients with pulmonary coccidiomycosis usually develop an upper lobe infiltrated associated with hilar or mediastinal adenopathy, with life-threatening clinical manifestations only when severely immunocompromised. The diagnosis is proven by culture of *Coccidioides* spp. from any clinical site, although Enzyme Immuno Assay (EIA), immunodiffusion, complement fixation (CF) and serological testing may be useful and more easily accessible [136].

Treatment with fluconazole or itraconazole should be given to all immunocompromised patients or with cardiopulmonary comorbidities, such as severe COVID-19, in order to reduce the risk of extra-pulmonary dissemination. Severe disease should be treated with ampohotericine B formulation, followed by a triazole [136,137].

The diagnosis of histoplasmosis may be challenging, due to the wide range of its clinical manifestations. The sensitivity of tissue examinations, conventional blood cultures and serological testing depends on the severity of the immunosuppression of the patient [136].

All immunocompromised patients and those with progressive disseminated disease or concomitant pulmonary disease should be treated.

Treatment with liposomal amphotericin B, compared with amphotericin B deoxycholate, has been shown to provide a survival benefit in patients with HIV and disseminated histoplasmosis, whereas voriconazole is not recommended. After induction therapy, maintenance treatment with itraconazole is usually recommended to be continued for at least 1 year, only then re-initiating any pharmacological immunosuppression [136].

## 7. Conclusions

Two years after the pandemic outbreak, a growing number of observational studies and case reports is still showing that COVID-19 clinical course can be often complicated by secondary respiratory fungal infections. Many factors hinder reliable data about the epidemiology of these coinfections, including the lack of an endorsed case definition and the difficulty of getting a histopathological confirmation. CAPA is certainly the most frequent fungal coinfection in COVID-19 patients, at least in European countries, probably contributing to increase the mortality rate of critically ill patients. Nevertheless, a fair number of non-*Aspergillus* coinfections have been observed and they should always be considered in the diagnostic algorithm, especially in patients belonging to the high-risk groups described above. The apparent rarity of the diagnosis of CAM, compared with the likely overdiagnosis of CAPA, may be due to the non-availability of biomarkers for CAM, such as 1,3-β-D-glucan and galactomannan available instead for CAPA, making its diagnosis easier and probably excessive. Indeed, invasive fungal respiratory infections in COVID-19 patients are associated to worse outcomes and increased mortality rates, explaining the detrimental importance of an early diagnosis and a consequent appropriate antifungal treatment. On the other hand, prophylaxis in COVID-19 patients with antifungal drugs is currently not supported by reliable data and should be considered only after more solid data from prospective clinical trials. Further studies on the physiopathology of invasive respiratory fungal infections are needed with the aim at improving diagnostic and therapeutic approaches, useful not only in the context of the present pandemic, but also in the other well-known high-risk conditions.

## Figures and Tables

**Table 1:** Treatment of principal invasive respiratory fungal infections.

	First Choice	Alternatives	Comments
CAPA	Voriconazole (Day 1 6 mg/kg bid, from day 2 4 mg/kg bid/day) Isavuconazole (Day 1–2 200 mg tid/day, from day 3 200 mg/day)	Liposomial amphotericin B (3–5 mg/kg/day) Echinocandins (Day 1 70 mg, from day 2 50 mg/day) Posaconazole (Day 1 300 mg bid, from day 2 300 mg/day)	Consider voriconazole drug-drug interaction with COVID-19 therapies (i.e., dexamethasone and remdesivir). Recommended weekly therapeutic drug monitoring for voriconazole (plasma trough concentration 2–6 mg/L) and posaconazole (plasma trough concentration 1 mg/L). Liposomial amphotericin B can be considered for initial therapy in suspected or proven azole resistant. Echinocandins not recommended as monotherapy.
CAM	Liposomal amphotericin B (5–10 mg/kg/day)	Isavuconazole (Day 1–2 200 mg tid/day, from day 3 200 mg/day) Posaconazole (Day 1 300 mg bid, from day 2 300 mg/day IV)	Surgical debridement of primary focus is strongly recommended. Isavuconazole and posaconazole may be considered as step-down therapy once disease is controlled and susceptibility confirmed.
PCP	Trimethoprim-sulfamethoxazole (15–20 mg TMP/kg/day divided q6–8h)	Pentamidine (4 mg/kg/day)	Routine adjunctive corticosteroids in non-HIV patients is not recommended and may be used on an individual patient basis.
Cryptococcosis	Fluconazole (400 mg/day)	Itraconazole (200 mg bid/day) Voriconazole (200 mg bid/day) Posaconazole (Day 1 300 mg bid, from day 2 300 mg/day)	The alternative regimens are indicated for mild-to-moderate pulmonary disease. For more severe pulmonary disease or disseminated disease: Induction therapy: Liposomal AmB (3–4 mg/kg/day) + flucytosine 25 mg/kg q6 per day for 4 weeks; Consolidation therapy: fluconazole (400–800 mg/day) for 8 weeks; Maintenance therapy: fluconazole (200 mg/day) for 6–12 months. Recommended perform a lumbar puncture to rule out CNS disease, particularly in immunocompromised hosts.

CAPA: COVID-19 associated pulmonary aspergillosis; CAM: COVID-19 associated mucormycosis; PCP: *Pneumocystis jirovecii* pneumonia; bid: twice a day; tid: three times a day; CNS: central nervous system.

## Data Availability

Not applicable.
